# Human-Like Obstacle Avoidance Trajectory Planning and Tracking Model for Autonomous Vehicles That Considers the Driver’s Operation Characteristics

**DOI:** 10.3390/s20174821

**Published:** 2020-08-26

**Authors:** Qinyu Sun, Yingshi Guo, Rui Fu, Chang Wang, Wei Yuan

**Affiliations:** School of Automobile, Chang’an University, Xi’an 710064, China; sunqinyu@chd.edu.cn (Q.S.); furui@chd.edu.cn (R.F.); wangchang@chd.edu.cn (C.W.); yuanwei@chd.edu.cn (W.Y.)

**Keywords:** autonomous vehicle, obstacle avoidance, artificial potential field, model predictive control, human-like

## Abstract

Developing a human-like autonomous driving system has gained increasing amounts of attention from both technology companies and academic institutions, as it can improve the interpretability and acceptance of the autonomous system. Planning a safe and human-like obstacle avoidance trajectory is one of the critical issues for the development of autonomous vehicles (AVs). However, when designing automatic obstacle avoidance systems, few studies have focused on the obstacle avoidance characteristics of human drivers. This paper aims to develop an obstacle avoidance trajectory planning and trajectory tracking model for AVs that is consistent with the characteristics of human drivers’ obstacle avoidance trajectory. Therefore, a modified artificial potential field (APF) model was established by adding a road boundary repulsive potential field and ameliorating the obstacle repulsive potential field based on the traditional APF model. The model predictive control (MPC) algorithm was combined with the APF model to make the planning model satisfy the kinematic constraints of the vehicle. In addition, a human driver’s obstacle avoidance experiment was implemented based on a six-degree-of-freedom driving simulator equipped with multiple sensors to obtain the drivers’ operation characteristics and provide a basis for parameter confirmation of the planning model. Then, a linear time-varying MPC algorithm was employed to construct the trajectory tracking model. Finally, a co-simulation model based on CarSim/Simulink was established for off-line simulation testing, and the results indicated that the proposed trajectory planning controller and the trajectory tracking controller were more human-like under the premise of ensuring the safety and comfort of the obstacle avoidance operation, providing a foundation for the development of AVs.

## 1. Introduction

The vehicle active obstacle avoidance system is one of the core issues in the research of autonomous vehicle (AV) control [1,2]. A safe and reasonable obstacle avoidance trajectory planning in real time based on accurate obstacle information perception through multiple sensors can promote trajectory tracking technology, which can effectively improve the intelligent level of the autonomous system and reduce the frequency of traffic accidents [3,4,5]. As one of the key technologies of an active obstacle avoidance system for vehicles, the local trajectory replanning refers to designing a safe trajectory that enables AVs to promptly and accurately bypass obstacles based on global path planning [6]. Under the premise of satisfying multiple constraints, the designed trajectory should also comply with human drivers’ driving characteristics of obstacle avoidance. Therefore, active obstacle avoidance trajectory planning and control have become a difficulty in vehicle lateral control. Determining how to ameliorate the human-like degree of trajectory planning and tracking is the basis for achieving obstacle avoidance control, and is also an effective way for improving the safety and acceptability of AVs [7].

At present, the methods of local trajectory planning mainly include the fuzzy logic (FL) method, genetic algorithm (GA), neural network (NN) method, A* algorithm, rapidly exploring random tree (RRT), state-space trajectory-generation (ST), and artificial potential field (APF) method. The FL method is combined with the perception action of fuzzy control and replaces mathematical variables with linguistic variables [8]. Fuzzy control conditional statements describe the complex relationships between variables. Although the accuracy of the designed trajectory is improved, the FL algorithm itself lacks flexibility. The fuzzy rules and membership degree cannot change with the environment. The GA seeks the optimal solution by imitating the mechanism of selection and inheritance in nature, and then plans the trajectory through coding, crossover, mutation, and the construction of fitness function [9]. However, the programming implementation is relatively complex, and it is difficult to ensure real-time performance. The NN method uses a biologically-inspired NN method to establish an obstacle avoidance trajectory planning model. This method treats each grid as a neuron and the motion space of the vehicle is regarded as a topological NN [10]. Through training the grid, the parameters of the NN model can be adjusted to adapt to different scenarios. Therefore, the algorithm has good learning ability and stability, though the interpretability of the model is insufficient.

The heuristic search method represented by the A* algorithm has the characteristics of fast calculation speed and good flexibility [11]. Weight A* and ARA* accelerate the search direction to the target position by introducing and adjusting the weight value of the heuristic function [12]. A*-Connect adopts a bidirectional search strategy to obtain higher search efficiency [13]. Hybrid A* uses the variant of the A* search to plan trajectories that conform to kinematic constraints based on the three-dimensional motion state space, achieving local optimization through numerical nonlinear optimization [14]. By considering the vehicle motion direction information and the forward and backward movement patterns, a four-dimensional search space is constructed through this algorithm. The RRT method is an efficient planning method in multi-dimensional space [15]. RRT* adopts the tree node in the neighborhood with the lowest total generation value as the parent node, and reconnects the tree nodes in the neighborhood in each iteration, allowing the path on the random tree to always be progressively optimal. Informed-RRT* establishes an ellipsoid with the initial point and the target point as the focus [16]. This ellipsoid is employed as the search domain to gradually optimize the path, and the area of the ellipsoid decreases during the search process. Finally, the ellipsoid converges into the optimal path. Reachability-guided RRT reduces the sensitivity of systems with different constraints to random sampling for measurements in the extended tree, and it is only possible to select the node when the distance from the sampling point to the node is greater than the distance from the sampling point to the accessible set of the node [17]. Continuous-curvature RRT adopts a target-biased sampling strategy, a node connection mechanism based on a reasonable metric function, and a post-processing method that satisfies vehicle motion constraints to improve the speed and quality of planning [18].

The APF method was first proposed by Khatib in the study regarding the obstacle avoidance trajectory planning of robots [19]. It has the characteristics of a small amount of calculation, short planning time, and high execution efficiency. In recent years, it has been gradually applied to the local trajectory planning of intelligent vehicles [20]. The basic theory is to establish an obstacle repulsive potential field and a target point gravitational potential field through the sensor’s perception of the environment, as well as to find the descending path of the total potential field as the obstacle avoidance path in the compound potential field [21]. However, the traditional APF method has problems such as local minimum points, and the obstacle avoidance trajectory planning for the robot does not consider the robot’s own size range and boundary environment. The intelligent vehicle’s obstacle avoidance is also constrained by the size of the vehicle and obstacles and the road boundary [22]. In addition, the planning trajectory should also meet the actual dynamic and kinematic constraints. Therefore, the traditional APF method needs to be improved to satisfy the need for trajectory planning for AVs. Tokson et al. [23] used the gradient descent algorithm of the potential field to find an effective path in the potential energy field, and added repulsive potential energy when the vehicle failed into a minimum point, to construct a modified potential field to make the controlled object continue to move towards the target point. Bounini et al. [24] proposed the concept of the steering potential field. The steering potential field is established by issuing steering commands to intelligent vehicles through remote control stations or vehicle-mounted navigation systems. Then, the obstacle repulsion potential field is established according to obstacle data. Raja et al. [25] introduced a gradient function in the traditional APF method, which was composed of gravity, repulsion, tangential force, and gradient force according to a certain weight to form a modified potential field function. The gradient force is the function of vehicle yaw and pitch angle at a specific position, which ensures that the vehicle does not drive in the direction of high gradient force, thus obtaining the expected obstacle avoidance trajectory. Zhang et al. [26] employed the elliptic distance to replace the actual distance in the traditional repulsion potential field, and comprehensively considered the influence of lane lines, obstacles, and the road boundary potential energy field on vehicles to obtain a smoother local obstacle avoidance trajectory. Kenealy et al. [27] proposed an enhanced space-based potential field model to realize through the exploration of complex environments for autonomous robots.

Trajectory tracking has been the subject of numerous empirical studies that have investigated different control algorithms to establish an autonomous control model. The commonly used tracking control algorithms mainly include the proportion integration differentiation (PID) control algorithm, optimal preview control (OPC) algorithm, fuzzy control algorithm, sliding mode control (SMC) algorithm, linear quadratic regulator (LQR) control algorithm, and model predictive control (MPC) algorithm. The traditional PID control mainly relies on adjusting the gains of the three parts of the proportional unit, integral unit, and differential unit to set its characteristics [28]. This algorithm is simple and easy to operate, but has poor adaptability in trajectory tracking under complex conditions. The OPC algorithm sets a preview point on the forward road of the vehicle, and realizes the tracking control of the expected trajectory by reducing the lateral deviation between the preview point and the expected road center line [29]. Liu et al. [30] improved the preview distance based on longitudinal speed and steering angle feedback, and designed a trajectory tracking controller according to the preview error model to make the change of steering angle more stable. Park et al. [31] applied the proportion integration control in the lateral offset to reduce tracking error and alleviate the impact of preview distance with velocity change. The fuzzy control algorithm is mainly divided into three parts: input fuzzy, fuzzy reasoning, and defuzzification [32]. The trajectory tracking is realized by designing different membership functions. The increment of the front wheel angle was taken as the output of the controller for the controller designed by Trabia et al. [33], which included a steering fuzzy module and an obstacle avoidance fuzzy module. This algorithm reduced the computation of the controller. The SMC algorithm, also known as variable structure control, can dynamically change based on the current state of the system to achieve the goal of gradually stabilizing to the equilibrium point according to the predetermined state trajectory [34]. The MPC algorithm has advantages in dealing with linear and nonlinear systems with constraints [35]. Kong et al. [36] designed MPC controllers based on vehicle kinematics and dynamics, and implemented tests to compare the prediction errors of the two controllers. The results demonstrated that the MPC controller based on kinematics had better performance and less computation under the low speed condition. Zanon et al. [37] combined the nonlinear MPC with the moving vision estimation method to solve the problem of poor trajectory tracking accuracy on the road with a low friction coefficient.

How to improve the real-time performance and human-like degree is the difficulty and kernel of trajectory planning research. The trajectory planning model based on machine learning and deep learning algorithm can take into account the above two key points, but the inexplicability of the model still cannot be effectively solved. The heuristic search method represented by the A* algorithm has the characteristics of fast calculation speed. However, there are some differences between the planned trajectory and the actual driving trajectory derived from human drivers, since this type of algorithm relies more on the data processing method of computer and lacks a mechanism model similar to driver behavior. The traditional APF model could simulate the obstacle avoidance behavior of drivers, but how to prompt the human-like degree and some defects of the algorithm still need further study. In the actual driving process, the driver will control the vehicle in advance through preview behavior, and MPC algorithm can simulate the preview behavior of the driver by adjusting the prediction time domain. Existing research combines the APF trajectory planning model with the MPC algorithm to achieve obstacle avoidance. Due to the complexity of the vehicle dynamic model and considering the real-time requirements, the prediction time domain in the MPC algorithm cannot set too large. In addition, the vehicle kinematic model is frequently ignored in the control models. On the one hand, the human-like degree of the obstacle avoidance control would be weakened, and on the other hand, the comfort and smoothness of the planned trajectory would be influenced.

To address the deficiencies in the obstacle avoidance trajectory planning model based on the APF algorithm and the trajectory tracking model based on the MPC algorithm, a modified APF algorithm was proposed in the present research by establishing a road boundary repulsion potential field and an obstacle repulsion potential field with variable parameter. To make the planned obstacle avoidance trajectory meet the vehicle kinematics constraints and ameliorate the human-like degree, the APF algorithm was combined with the MPC algorithm to construct the obstacle avoidance trajectory replanning controller. Considering that there are many kinds of constraints during vehicle lateral control and for the sake of guaranteeing the real-time capability, accuracy, and robustness of the trajectory tracking control algorithm at different speeds, a linear time-varying model predictive trajectory tracking controller was established based on linearizing the vehicle monorail dynamic model. The controller on the basis of MPC determined the vehicle front wheel angle as the control variable, and multiple constraints for the vehicle dynamics and kinematics were combined to design the objective function that can achieve the requirements of fast and accurate tracking of the desired trajectory. In addition, this work implemented driver obstacle avoidance experiments under different speeds based on a driving simulator with six degrees of freedom to ensure that the established trajectory planning model was consistent with a human driver’s obstacle avoidance characteristics; that is, the planning trajectory was similar to the driver operation trajectory. Two pivotal parameters in the APF algorithm were determined to enhance the human-like degree of planned trajectory and the trajectory characteristics derived from human drivers were extracted to provide a basis for the parameters design of the proposed trajectory planning model for AVs. Finally, the co-simulation model based on CarSim/Simulink was established for the off-line simulation testing of the obstacle avoidance trajectory planning controller and the trajectory tracking controller designed in this study.

**Figure 1:**
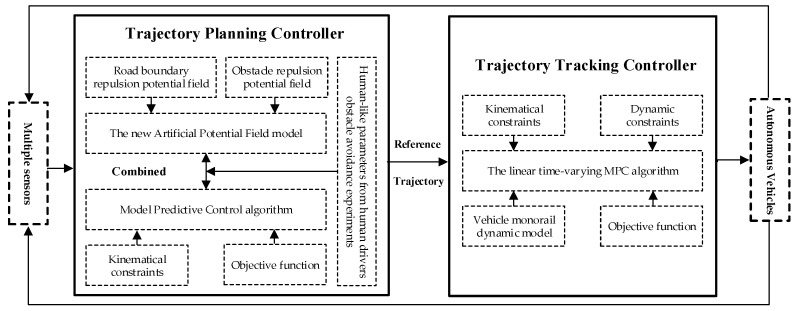
Human-like obstacle avoidance system framework for AVs.

The remainder of the paper is organized as follows. Section 2 details the obstacle avoidance trajectory planning model based on the APF algorithm and the MPC algorithm. Section 3 provides detailed information on the trajectory tracking model based on the linear time-varying MPC algorithm. Section 4 presents the experimental design, process, equipment, and feature analysis of the human driver’s obstacle avoidance trajectory. The co-simulation results of the proposed trajectory planning controller and trajectory tracking controller are introduced in Section 5. Finally, conclusions are presented in Section 6. The main framework of this study is presented in Figure 1. 

## 2. Obstacle Avoidance Trajectory Planning Model

### 2.1. Traditional Artificial Potential Field Model

**Figure 2:**
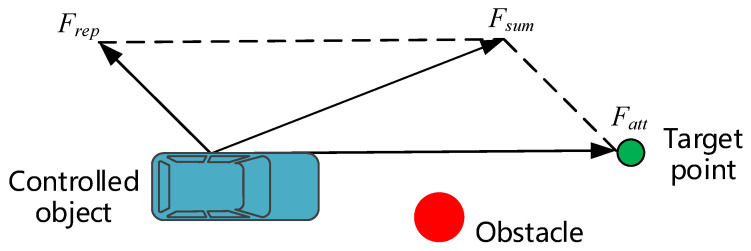
Model of the traditional APF algorithm.

Khatib first proposed the APF algorithm in 1986. The basic idea of this algorithm is to virtualize the motion of the controlled object in the environment as a forced motion of particles in the artificial virtual force field [38]. The obstacle exerted a repulsive force on the controlled object, and the target point exerted a gravitational force on the controlled object. The controlled object moved toward the combined force of the repulsive force and the gravitational force, as shown in Figure 2. In the figure, $F_{rep}$ is the repulsive force generated by the obstacle, $F_{att}$ is the gravitational force generated by the target point, and $F_{sum}$ is the resultant force. The distance between the controlled object and the obstacle and the target point mainly determines the magnitude of the repulsive force and gravitational force. The smaller the distance between the controlled object and the obstacle, the greater the repulsive force. Further, the greater the distance between the controlled object and the target point, the greater the gravity.

In the traditional APF algorithm, the controlled object is reduced to a particle, and its motion space is regarded as a two-dimensional Euclidean space. Assuming that the coordinate of the controlled object X in space is (x,y) and the target point $X_{goal}$ coordinate is $\left(x_{goal},y_{goal}\right)$, the gravitational field function of the controlled object in space is defined as a quadratic function related to the position of the controlled object and the target point:

(1) $$U_{att}\left(X\right)=\frac{1}{2}k_{g}\rho_{g}{}^{2},$$

where $k_{g}$ is the gain coefficient of the gravitational potential field, $\rho_{g}$ is the relative distance between the controlled vehicle and the target point, the value is the vector, and the direction is the controlled vehicle points to the target point.

The gravitational force on the controlled object can be obtained by calculating the negative gradient of the gravitational potential field:

(2) $$F_{att}\left(X\right)=-\nabla U_{att}\left(X\right)=-k_{g}{\vec{u}}_{g},$$

where ${\vec{u}}_{g}$ is the unit vector where the controlled object points to the target point.

Assuming that the coordinates of the obstacle $X_{obs}$ in the space is $\left(x_{obs},y_{obs}\right)$, the repulsive force field function on the controlled object is defined as:

(3) $$U_{rep}\left(X\right)=\{\begin{array}{c} \frac{1}{2}k_{o}{\left(\frac{1}{\rho_{ob}}-\frac{1}{\rho_{o}}\right)}^{2},\rho_{ob}\leq \rho_{o}\\ 0,\rho_{ob}>\rho_{o} \end{array}$$

where $k_{o}$ is the repulsive potential field coefficient, $\rho_{ob}$ is the distance constant between the controlled object and the obstacle, and $\rho_{o}$ is the influence range of the repulsive potential field of the obstacle. When $\rho_{ob}>\rho_{o}$, the controlled object is not affected by the repulsive force of the obstacle. 

The repulsive force on the controlled object can be obtained by calculating the negative gradient of the repulsive potential field:

(4) $$F_{rep}\left(X\right)=-\nabla U_{rep}\left(X\right)=\{\begin{array}{c} k_{o}{\left(\frac{1}{\rho_{ob}}-\frac{1}{\rho_{o}}\right)}^{2}{\vec{u}}_{ob},\rho_{ob}\leq \rho_{o}\\ 0,\rho_{ob}>\rho_{o} \end{array}$$

where ${\vec{u}}_{ob}$ is the unit vector where the obstacle points to the controlled object.

Therefore, the combined force of the controlled object when moving in the force field space is:

(5) $$F_{sum}\left(X\right)=-\nabla U_{sum}\left(X\right)=F_{att}\left(X\right)+\overset{n}{\underset{i=1}{\sum }}F_{rep,i}\left(X\right),$$

where *n* is the number of obstacles. 

The traditional APF algorithm has the following problems when it is used to plan the local obstacle avoidance trajectory of vehicles [39].

(1) Lack of road boundary constraints. The algorithm only considers the passability of obstacle avoidance trajectories, and does not consider the road boundary constraints during vehicle driving.

(2) The goal may be unreachable. When there is an obstacle near the target point, the repulsive force of the vehicle when approaching the target point is greater than the gravitational force, so that the controlled object cannot reach the target point.

(3) The controlled object may come to a deadlock. There may be a situation where the controlled object receives the same repulsive force and gravity at a certain point, resulting in the controlled object being unable to continue to advance. 

### 2.2. Modified Artificial Potential Field Model

In order to solve the above deficiencies in the traditional APF algorithm, a modified APF model is proposed through establishing the road boundary repulsive potential field, ameliorating the obstacle potential field, and combining with the MPC algorithm. 

#### 2.2.1. Road Boundary Repulsive Potential Field

**Figure 3:**
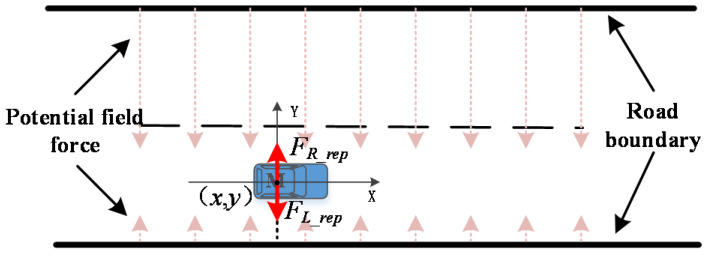
Schematic diagram of the road boundary repulsive potential field force.

Road boundary repulsive potential field is established on the basis of the lane boundary, which is used to limit the driving area of vehicles to ensure that vehicles continue to drive along the center line of the lane after obstacle avoidance, and the vehicle body would not exceed the road boundary during the process of turning and avoiding obstacles. The established road boundary repulsive potential field is presented in Figure 3. The repulsive potential field generates a force based on the road boundary in the direction of the vehicle, and the repulsive force only takes the lateral force component of the earth coordinate system. The value of the road boundary repulsion is inversely proportional to the relative distance between the vehicle and boundary. The smaller the relative distance, the greater the repulsion, and the larger the relative distance, the smaller the repulsion. 

When there is no obstacle in the lane, the vehicle travels along the center line of the right lane under the action of the repulsion potential field of the road boundary. Considering the size of the vehicle, the road boundary repulsive potential field model is established as follows:

(6) $$\{\begin{array}{c} U_{L\_rep}\left(X\right)=\frac{1}{2}k_{L\_rep}{\left(\frac{1}{\rho_{L{\_}_{rep}-}\frac{w_{v}}{2}}\right)}^{2}\\ U_{R\_rep}\left(X\right)=\frac{1}{2}k_{R\_rep}{\left(\frac{1}{\rho_{R{\_}_{rep}-}\frac{w_{v}}{2}}\right)}^{2} \end{array}$$

where $k_{L\_rep}$ and $k_{R\_rep}$ are the repulsive potential field coefficients of the left and right road boundaries, respectively; $w_{v}$ is the lateral width of the vehicle, and $\rho_{L{\_}_{rep}}$ and $\rho_{R{\_}_{rep}}$ are the shortest distances between the center of mass of the vehicle and the boundary of the left and right lanes, respectively. 

The repulsive force on the controlled object can be obtained by calculating the negative gradient of the road boundary repulsive potential field:

(7) $$\{\begin{array}{c} F_{L\_rep}\left(X\right)=k_{L\_rep}\left(\frac{1}{\rho_{L{\_}_{rep}-}\frac{w_{v}}{2}}\right){\left(\frac{1}{\rho_{L{\_}_{rep}}}\right)}^{3}{\vec{a}}_{Lv}\\ F_{R\_rep}\left(X\right)=k_{R\_rep}\left(\frac{1}{\rho_{R{\_}_{rep}-}\frac{w_{v}}{2}}\right){\left(\frac{1}{\rho_{R{\_}_{rep}}}\right)}^{3}{\vec{a}}_{Rv} \end{array}$$

where ${\vec{a}}_{Lv}$ and ${\vec{a}}_{Rv}$ are the unit vector where the road boundaries point to the controlled object.

#### 2.2.2. Obstacle Repulsive Potential Field

**Figure 4:**
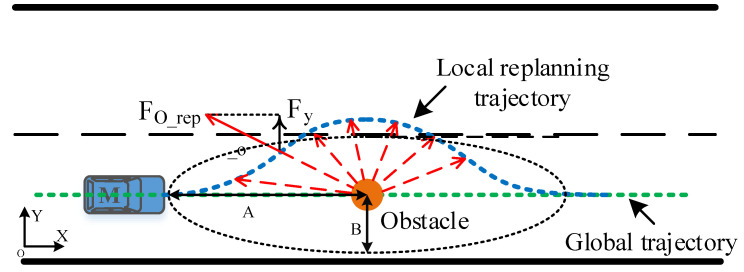
Schematic diagram of the road boundary repulsive potential field force.

The circular repulsion field of the traditional APF does not satisfy the requirements of the actual vehicle obstacle avoidance trajectory according to the human driver experience, and it is difficult to meet the requirements of steering smoothness in the trajectory planning, resulting in a decrease of ride comfort. Therefore, the scope of action of the potential field was modified in this work, and the longitudinal action distance of the obstacle repulsion potential field was increased, so that the vehicle can correct the direction in advance to avoid obstacles; the lateral action distance was reduced to prevent the vehicle from driving out of the lane during obstacle avoidance. Figure 4 illustrates the schematic diagram of the obstacle repulsive potential field. The longitudinal and lateral acting distances of the repulsive potential field of an obstacle were defined as A and B, respectively. The scope of action of the repulsive field $\rho_{o}$ can be rewritten as:

(8) $$\rho_{o}\in \frac{{\left(x-x_{obs}\right)}^{2}}{A^{2}}+\frac{{\left(y-y_{obs}\right)}^{2}}{B^{2}}$$

Considering that the obstacle avoidance process is similar to the lane change process, the force exerted by the obstacle repulsive potential field on the vehicle can only be retained in the lateral component under the geodetic coordinate system to avoid the vehicle coming to a deadlock. The repulsive direction of the obstacle to the vehicle is upward when the vehicle enters the obstacle repulsive potential field. At this time, the vehicle will turn to the left for avoidance. During this process, the repulsive potential energy increases with the decrease of the relative distance between the vehicle and the obstacle, thus forcing the vehicle to drive away from the obstacle.

The obstacle repulsive potential field is established with an obstacle as the center of the potential energy. Within the scope of action of the repulsive potential field of an obstacle, it exerts a repulsive force on the vehicle to keep the vehicle away from the obstacle. In the traditional APF model, the gravitational force is less than the repulsive force when the vehicle reaches the target point, which will lead to the problem of unreachable target. Therefore, an adjustment factor $R_{d}^{m}$ is added to the obstacle repulsive potential field. In this way, the relative distance between the vehicle and the target point $R_{d}$ is supplemented in the modified obstacle repulsive potential field. Hence the repulsive force and the gravitation force are reduced to zero at the same time only when the vehicle reaches the target point, so that the problem of unreachable target is solved. The modified obstacle repulsion potential field function is shown as follows:

(9) $$U_{o\_rep}\left(X\right)=\{\begin{array}{c} \eta_{rep}\left\{exp\left[-\frac{1}{2}\left(\frac{{\left(x-x_{obs}\right)}^{2}}{A^{2}}+\frac{{\left(y-y_{obs}\right)}^{2}}{B^{2}}\right)\right]\right\}R_{d}^{m},\;p_{o\_rep}^{n}\leq \rho_{o}\\ 0,p_{o\_rep}^{n}>\rho_{o} \end{array}$$

where $R_{d}$ is the relative distance between the vehicle and the target point, m is constant, $\eta_{rep}$ is the repulsive potential field coefficient of the obstacle, $p_{o\_rep}^{n}$ is the distance between the vehicle and the *nth* obstacle, and $\rho_{o}$ is the range of action of the repulsive field. 

In addition, the vehicle may come to a deadlock when the repulsive force from other surrounding vehicles is equal to the gravitational force. In this case, the value of m in the adjustment factor will gradually increase from 0 until the force balance is broken, so that the vehicle could jump out of the local minimum and then the value of m would return to the original value. Within the scope of the obstacle, the repulsive force on the controlled object can be obtained by calculating the negative gradient of the obstacle repulsive potential field:

(10) $$F_{o\_rep}\left(X\right)=\{\begin{array}{c} -\eta_{rep}{\left(\frac{{\left(x-x_{obs}\right)}^{2}}{A^{2}}+\frac{{\left(y-y_{obs}\right)}^{2}}{B^{2}}\right)}^{2}R_{d}^{m}{\vec{a}}_{ov},\;p_{o\_rep}^{n}\leq \rho_{o}\\ 0,p_{o\_rep}^{n}>\rho_{o} \end{array},$$

where ${\vec{a}}_{ov}$ is the unit vector where the obstacle points to the controlled object. 

### 2.3. Model Prediction Algorithm With Trajectory Planning

**Figure 5:**
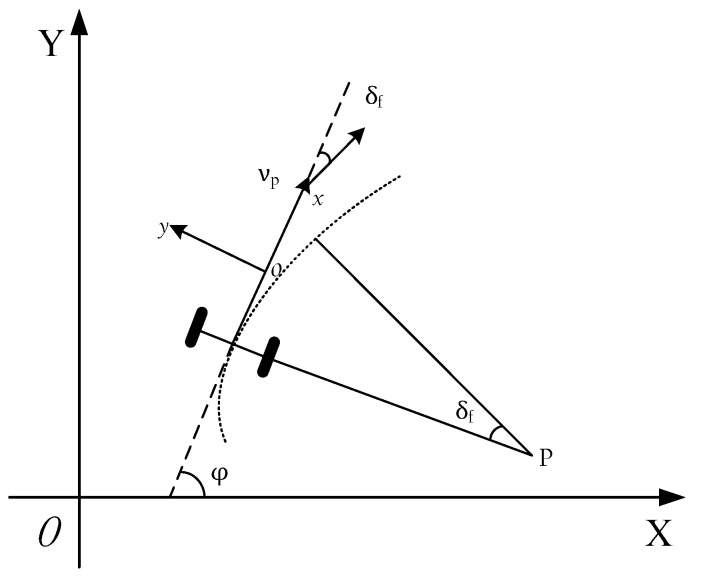
The vehicle kinematics model.

To ensure that the planning trajectory of the modified APF model is practical and can satisfy the kinematic constraints of the vehicle, the MPC algorithm was combined with the modified APF model and a reasonable objective function was constructed to minimize the deviation between the planning trajectory of the modified APF model and the predicted trajectory of the MPC algorithm. Due to the low real-time requirement of the planning layer, the adoption of a relatively simple point mass model can fully meet the requirements of re-planning. Therefore, as shown in Figure 5, the steering motion model was established with XOY as the geodetic coordinate system and xoy as the vehicle coordinate system. 

The vehicle kinematics model can be expressed as follows:

(11) $$\{\begin{array}{c} \dot{X}=\dot{x}cos\left(\varphi \right)-\dot{y}sin\left(\varphi \right)\\ \dot{Y}=\dot{x}sin\left(\varphi \right)-\dot{y}cos\left(\varphi \right)\\ \begin{array}{c} \dot{\varphi }=\frac{\ddot{y}}{\dot{x}}\\ \ddot{x}=0 \end{array} \end{array}$$

where $\dot{x}$ and $\dot{y}$ represent the longitudinal and lateral speeds in the vehicle coordinate system, respectively; $\ddot{x}$ and $\ddot{y}$ correspondingly represent the longitudinal and lateral accelerations in the vehicle coordinate system; φ and $\dot{\varphi }$ represent the yaw angle and yaw rate of the vehicle, respectively; $\dot{X}$ and $\dot{Y}$ correspondingly represent the longitudinal and lateral speeds in the geodetic coordinate system. 

This article only considers the obstacle avoidance strategy of the vehicle at constant speed, so the longitudinal acceleration is set to zero. Five discrete state variables were determined as $X=\left[\dot{x},\dot{y},\varphi ,X,Y\right]$, and the lateral acceleration was selected as the control variable $v=\left[\ddot{y}\right]$. Then the state equation can be expressed as:

(12) $$\dot{X}\left(t\right)=f\left(X\left(t\right),v\left(t\right)\right)$$

Using Taylor expansion and first-order difference quotient to linearize and discretize Equation (13), the linear time-varying model can be obtained as follows:

(13) $$\tilde{X}\left(k+1\right)={\tilde{A}}_{p}\left(k\right)\tilde{X}\left(k\right)+{\tilde{B}}_{p}\left(k\right)\tilde{v}\left(k\right)$$

where ${\tilde{A}}_{p}\left(k\right)=\left[\begin{array}{ccc} 1 & 0 & -\dot{x}sin\left(\varphi \right)T\\ 0 & 1 & \dot{x}cos\left(\varphi \right)T\\ 0 & 0 & 1 \end{array}\right]$, ${\tilde{B}}_{p}\left(k\right)=\left[\begin{array}{cc} cos\left(\varphi \right)T & 0\\ sin\left(\varphi \right)T & 0\\ T\tan\left(\delta_{f}\right)T/l & \dot{x}T/lcos^{2}\left(\delta_{f}\right) \end{array}\right]$, T is the sampling time, l is the wheel base, and $\delta_{f}$ is the front wheel angle. 

The control objective in the trajectory planning layer is to minimize the deviation between the planning trajectory of the modified APF model and the predicted trajectory of the MPC algorithm under the premise of ensuring the smooth and comfortable driving of vehicles. Therefore, the objective function of trajectory planning is defined as follows:

(14) $$J_{p}\left(k\right)=\overset{N_{pp}}{\underset{j=1}{\sum }}\|\eta_{p}\left(k+j|t\right)-\eta_{ref}{\left(k+j|t)\right\|}_{Q_{p}}^{2}+\overset{N_{pc}-1}{\underset{j=1}{\sum }}\|\Delta \tilde{v}{\left(k+j|t\right)\|}_{R_{p}}^{2},$$

where $Q_{p}$ and $R_{p}$ are the weight matrixes, $\eta_{ref}$ is the planning trajectory of the modified APF model, $\eta_{p}$ is the predicted trajectory of the MPC algorithm, and $N_{pp}$ and $N_{pc}$ are, respectively, the prediction step size and control step size of the MPC controller. 

Then the output can be expressed as:

(15) $$\tilde{Y}\left(k\right)=\psi_{p}\tilde{X}\left(k\right)+\Theta_{p}\Delta \tilde{v}\left(k\right),$$

(16) $$\eta_{p}\left(k\right)={\tilde{C}}_{p}\tilde{X}\left(k\right),$$

where, $\tilde{Y}\left(k\right)=\left[\begin{array}{c} \begin{array}{c} \eta_{p}\left(k+1|k\right)\\ \eta_{p}\left(k+2|k\right)\\ \vdots \end{array}\\ \eta_{p}\left(k+N_{pc}|k\right)\\ \begin{array}{c} \vdots \\ \eta_{p}\left(k+N_{pp}|k\right) \end{array} \end{array}\right]$, $\psi_{p}=\left[\begin{array}{c} \begin{array}{c} {\tilde{C}}_{p}{\tilde{B}}_{p}\\ {\tilde{C}}_{p}{\tilde{B}}_{p}^{2}\\ \vdots \end{array}\\ {\tilde{C}}_{p}{\tilde{B}}_{p}^{N_{pc}}\\ \begin{array}{c} \vdots \\ {\tilde{C}}_{p}{\tilde{B}}_{p}^{N_{pp}} \end{array} \end{array}\right]$, ${\tilde{C}}_{p}=\left[\begin{array}{c} 0\;0\;1\;0\;0\;0\;00\\ 0\;0\;0\;0\;1\;0\;00 \end{array}\right]$, $\Theta_{p}=(\begin{array}{c} \begin{array}{cccc} {\tilde{C}}_{p}{\tilde{B}}_{p} & 0 & 0 & 0 \end{array}\\ \begin{array}{cccc} {\tilde{C}}_{p}{\tilde{A}}_{p}{\tilde{B}}_{p} & {\tilde{C}}_{p}{\tilde{B}}_{p} & 0 & 0 \end{array}\\ \begin{array}{cccc} \vdots & \vdots & \ddots & \vdots \end{array}\\ \begin{array}{cccc} {\tilde{C}}_{p}{\tilde{A}}_{p}^{N_{pc}}{\tilde{B}}_{p} & {\tilde{C}}_{p}{\tilde{A}}_{p}^{N_{pc}-1}{\tilde{B}}_{p} & \cdots & {\tilde{C}}_{p}{\tilde{A}}_{p}{\tilde{B}}_{p} \end{array}\\ \begin{array}{cccc} \vdots & \vdots & \ddots & \vdots \end{array}\\ \begin{array}{cccc} {\tilde{C}}_{p}{\tilde{A}}_{p}^{N_{pp}-1}{\tilde{B}}_{p} & {\tilde{C}}_{p}{\tilde{A}}_{p}^{N_{pp}-2}{\tilde{B}}_{p} & \cdots & {\tilde{C}}_{p}{\tilde{A}}_{p}^{N_{pp}-N_{pc}-1}{\tilde{B}}_{p} \end{array} \end{array}).$

It is also necessary to append obstacle avoidance constraints and limit the control variables for the sake of ensuring that the planning trajectory is practical. The obstacle avoidance constraints are divided into road constraints and obstacle constraints:

(17) $$\{\begin{array}{c} \rho_{L\_rep}>\frac{w_{v}}{2}\\ \rho_{R\_rep}>\frac{w_{v}}{2}\\ \rho_{obs}{}^{n}>1.2w_{obs} \end{array}$$

where $w_{obs}$ is the width of the obstacle. 

In addition, the lateral acceleration of the vehicle is mainly provided by the lateral force of the tire, so it must meet the limit of tire adhesion:

(18) $$\ddot{y}\in \left[-\mu g,\;\mu g\right]$$

where μ is the coefficient of road adhesion, and g is the acceleration of gravity. 

Combining Equations (15), (18) and (19), the trajectory planning model can be expressed as:

(19) $$\{\begin{array}{c} \begin{array}{c} min\overset{N_{pp}}{\underset{j=1}{\sum }}\|\eta_{p}\left(k+j|k\right)-\eta_{ref}{\left(k+j|k\right)\|}_{Q_{p}}^{2}+\overset{N_{pc}-1}{\underset{j=1}{\sum }}\|\Delta \tilde{\upsilon }{\left(k+j|k\right)\|}_{R_{p}}^{2}\\ st.\;\;\;\;\;\rho_{L\_rep}>\frac{w_{v}}{2} \end{array}\\ \rho_{L\_rep}>\frac{w_{v}}{2}\\ \begin{array}{c} \rho_{obs}{}^{n}>1.2w_{obs}\\ -\mu g\leq \ddot{y}\leq \mu g \end{array} \end{array}$$

## 3. Obstacle Avoidance Trajectory Tracking Model

The MPC algorithm can use the dynamic prediction model to obtain the future vehicle state in a limited time domain based on the current vehicle motion state. This method has a strong ability to deal with multi-objective constraints [40]. In this work, a linear time-varying MPC controller was established to track the trajectory from the obstacle avoidance trajectory planning model.

### 3.1. Vehicle Dynamic Model

Considering that the longitudinal speed remains unchanged and only the front wheel angle is controlled during obstacle avoidance, the following assumptions are made in the modeling process.

(1) The lateral forces and slip angles on the left and right tires of the vehicle are symmetric and equal in the vehicle coordinate system.

(2) The test sections are all flat roads, ignoring the influence of slope and other factors on the vertical movement of vehicles.

(3) The front wheel angle is small, and the lateral force of the tire is approximately linear with the slip angle of the tire. 

(4) The influence of the suspension system, transmission system, air resistance, and the longitudinal and lateral coupling force of the tire is ignored. 

**Figure 6:**
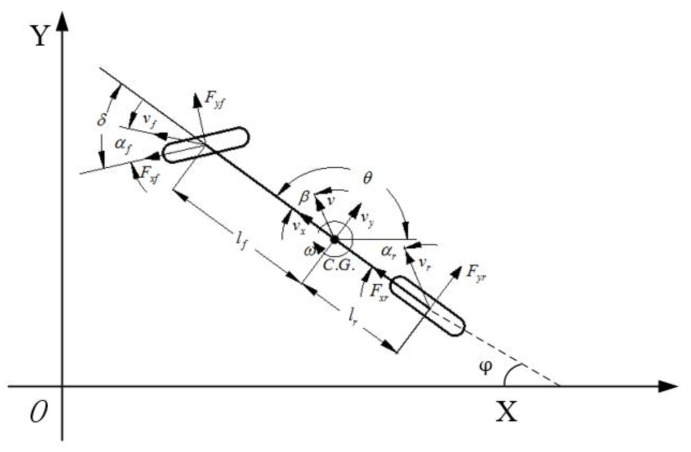
The vehicle monorail dynamics model.

The monorail dynamics model is established as shown in Figure 6, and the dynamic equation of the model can be described as follows:

(20) $$\{\begin{array}{c} \dot{X}=v_{x}cos\left(\varphi \right)-\left(v_{y}+l_{f}\dot{\varphi }\right)sin\left(\varphi \right)\\ \dot{Y}=v_{y}sin\left(\varphi \right)-\left(v_{y}+l_{f}\dot{\varphi }\right)cos\left(\varphi \right)\\ I_{z}\ddot{\varphi }=l_{f}F_{yf}-l_{r}F_{yr}\\ m{\dot{v}}_{x}=m{\dot{v}}_{x}\dot{\varphi }+F_{xf}+F_{xr}\\ m{\dot{v}}_{y}=-m{\dot{v}}_{y}\dot{\varphi }+F_{yf}+F_{yr} \end{array}$$

where $\dot{X}$ and $\dot{Y}$ represent the longitudinal and lateral speed in the geodetic coordinate system, $v_{x}$, $v_{y}$, and φ represent the longitudinal speed, lateral speed, and heading angle in the vehicle coordinate system, m represent vehicle mass, $l_{f}$ and $l_{r}$ represent the distance from the center of mass to the front and rear axles, $F_{xf}$, $F_{xr}$, $F_{yf}$, and $F_{yr}$ represents the longitudinal and lateral forces of the front and rear axles, and $I_{z}$ represent the moment of inertia. 

The state variables was determined as ${\hat{\xi }}_{c}\;=\;{\left[v_{x},v_{y},\varphi ,\dot{\varphi },X,Y\right]}^{T}$, and the control variable was selected as $v_{c}=\delta_{f}$. To satisfy the real-time requirements of the trajectory tracking controller when the vehicle is traveling at high speeds, the nonlinear dynamic model is linearized to obtain the linear time-varying equation:

(21) $${\hat{\xi }}_{c}=A_{c}\left(t\right){\dot{\xi }}_{c}\left(t\right)+B_{c}\left(t\right)v_{c}\left(t\right),$$

where $A_{c}\left(t\right)={\frac{\partial f\left({\dot{\xi }}_{c},v_{c}\right)}{\partial {\dot{\xi }}_{c}}|}_{{\dot{\xi }}_{c}\left(t\right),v_{c}\left(t\right)}$, and $B_{c}\left(t\right)={\frac{\partial f\left({\dot{\xi }}_{c},v_{c}\right)}{\partial v_{c}}|}_{{\dot{\xi }}_{c}\left(t\right),v_{c}\left(t\right)}$.

Using the first-order difference quotient to discretize Equation (22), the discrete state space expression can be obtained:

(22) $${\hat{\xi }}_{c}\left(k+1\right)=A_{c}\left(k\right){\dot{\xi }}_{c}\left(k\right)+B_{c}\left(k\right)v_{c}\left(k\right)$$

where $A_{c}\left(k\right)=I_{c}+TA_{c}\left(t\right)$, $B_{c}\left(k\right)=I_{c}+TB_{c}\left(t\right)$, $C_{c}=\left[\begin{array}{c} \begin{array}{ccc} 0 & 0 & \begin{array}{ccc} 1 & 0 & \begin{array}{cc} 0 & 0 \end{array} \end{array} \end{array}\\ \begin{array}{ccc} 0 & 0 & \begin{array}{ccc} 0 & 0 & \begin{array}{cc} 1 & 0 \end{array} \end{array} \end{array} \end{array}\right]$, and $I_{c}$ is unit matrix. 

### 3.2. Objective Function

To ensure that the trajectory tracking controller can promptly and smoothly track the expected trajectory, the following form of objective function is adopted:

(23) $$J_{c}\left(k\right)=\overset{N_{cp}}{\underset{i=1}{\sum }}\|\eta_{c}\left(k+i|t\right)-\eta_{pref}{\left(k+i|t\right)\|}_{Q_{c}}^{2}+\overset{N_{cc}-1}{\underset{i=1}{\sum }}\|\Delta U{\left(k+i|t\right)\|}_{R_{c}}^{2}+\rho \varepsilon^{2},$$

where $N_{cp}$ and $N_{cc}$ are the prediction step size and control step size of the controller respectively; $Q_{c}$ and $R_{c}$ are the weight coefficients, ε is the relaxation factor, ρ is the relaxation coefficient, and $\eta_{pref}$ is the expected trajectory from the trajectory planning controller. 

In Equation (24), the first item on the right side of the equal sign reflects the degree of tracking accuracy of the system; the second item is the constraint on the change of control quantity and increment of control quantity, reflecting the vehicle’s ability to maintain stability; the third item is the relaxation factor, which prevents the objective function from having no solution in the real-time calculation process. 

In the objective function, it is necessary to calculate the output of the vehicle in the predictive time domain based on the linear error model, and Equation (23) was converted into:

(24) $$\{\begin{array}{l} {\tilde{\xi }}_{c}(k+1|t)={\tilde{A}}_{c}\left(k\right){\dot{\xi }}_{c}(k|t)+{\tilde{B}}_{c}\left(k\right)\Delta v_{c}(k|t)\\ \eta_{c}\left(k|t\right)={\tilde{C}}_{c}{\dot{\xi }}_{c}(k|t)\;\;\;\;\;\;\;\;\;\;\;\;\;\;\;\;\;\;\;\;\;\;\;\;\;\;\;\;\;\;\;\;\;\;\;\;\;\;\;\;\;\;\;\;\;\;\; \end{array}$$

where ${\tilde{A}}_{c}\left(k\right)=\left[\begin{array}{cc} A_{c}\left(k\right) & B_{c}\left(k\right)\\ 0_{m\times n} & I_{m} \end{array}\right]$, ${\tilde{B}}_{c}\left(k\right)=\left[\begin{array}{c} B_{c}\left(k\right)\\ I_{m} \end{array}\right]$, ${\tilde{C}}_{c}=\left[\begin{array}{c} 0\;0\;1\;0\;0\;0\;00\\ 0\;0\;0\;0\;1\;0\;00 \end{array}\right]$, m is the dimension of state quantity, and n is the dimension of control quantity. 

To simplify the calculation, assume k=1,…,t+N−1, and the predicted output expression of the system can be deduced as follows:

(25) $$Y_{c}\left(t\right)=\psi_{c}{\tilde{\xi }}_{c}(t|t)+\Theta_{c}\Delta U_{c}(t|t)$$

where $Y\left(t\right)=\left[\begin{array}{l} \eta_{c}(t+1|t)\\ \eta_{c}(t+2|t)\\ \vdots \\ \eta_{c}(t+N_{cc}|t)\\ \vdots \\ \eta_{c}(t+N_{cp}|t) \end{array}\right]$, $\psi_{c}=\left[\begin{array}{l} {\tilde{C}}_{c}{\tilde{B}}_{c}\\ {\tilde{C}}_{c}{\tilde{B}}_{c}^{2}\\ \vdots \\ {\tilde{C}}_{c}{\tilde{B}}_{c}^{N_{cc}}\\ \vdots \\ {\tilde{C}}_{c}{\tilde{B}}_{c}^{N_{cp}} \end{array}\right]$, $\Delta U_{c}\left(t\right)=\left[\begin{array}{c} \Delta u(t|t)\\ \Delta u(t+1|t)\\ \vdots \\ \Delta u(t+N_{c}|t) \end{array}\right]$, and $\Theta_{c}=(\begin{array}{c} \begin{array}{cccc} {\tilde{C}}_{c}{\tilde{B}}_{c} & 0 & 0 & 0 \end{array}\\ \begin{array}{cccc} {\tilde{C}}_{c}{\tilde{A}}_{c}{\tilde{B}}_{c} & {\tilde{C}}_{c}{\tilde{B}}_{c} & 0 & 0 \end{array}\\ \begin{array}{cccc} \vdots & \vdots & \ddots & \vdots \end{array}\\ \begin{array}{cccc} {\tilde{C}}_{c}{\tilde{A}}_{c}^{N_{cc}}{\tilde{B}}_{c} & {\tilde{C}}_{c}{\tilde{A}}_{c}^{N_{cc}-1}{\tilde{B}}_{c} & \cdots & {\tilde{C}}_{c}{\tilde{A}}_{c}{\tilde{B}}_{c} \end{array}\\ \begin{array}{cccc} \vdots & \vdots & \ddots & \vdots \end{array}\\ \begin{array}{cccc} {\tilde{C}}_{c}{\tilde{A}}_{c}^{N_{cp}-1}{\tilde{B}}_{c} & {\tilde{C}}_{c}{\tilde{A}}_{c}^{N_{cp}-2}{\tilde{B}}_{c} & \cdots & {\tilde{C}}_{c}{\tilde{A}}_{c}^{N_{cp}-N_{cc}-1}{\tilde{B}}_{c} \end{array} \end{array}).$

By substituting Equation (26) into Equation (24), the complete objective function can be obtained. 

### 3.3. Constraint Condition

One of the advantages of the MPC controller is its ability to handle multiple target constraints. On the one hand, the design of the constraints in the optimization solution should match the mechanical design constraints of the vehicle steering mechanism. On the other hand, it should also satisfy the needs of vehicle smooth control. The vehicle dynamic constraints need to be considered in the actual trajectory tracking control process, and the specific constraints include the centroid slip angle constraint, tire slip angle constraint, and road adhesion condition. 

During the obstacle avoidance process, the front wheel angle and the increment of front wheel angle should satisfy the following constraints:

(26) $$\{\begin{array}{c} -25{}^{\circ}\leq \delta_{f}\leq 25{}^{\circ}\\ -0.47{}^{\circ}\leq \Delta \delta_{f}\leq 0.47{}^{\circ} \end{array}.$$

The centroid slip angle directly affects the vehicle’s driving stability and is an important reference index in vehicle stability control. The empirical formula of the centroid slip angle constraint is expressed as follows:

(27) −arctan(0.196μ)≤β≤arctan(0.196μ),

where μ is the coefficient of road adhesion. 

According to the relationship between the centroid slip angle and the front wheel angle, the tire slip angle can be expressed as:

(28) $$\{\begin{array}{c} \alpha_{f}=\frac{v_{y}+l_{f}\dot{\varphi }}{v_{x}}-\delta_{f}\\ \alpha_{r}=\frac{v_{y}+l_{r}\dot{\varphi }}{v_{x}} \end{array}.$$

There is a linear relationship between the slip angle and the corresponding lateral force of tire when the tire slip angle is relatively small. Hence, the front tire slip angle constraint can be expressed as:

(29) $$-6{}^{\circ}<\alpha_{f}<6{}^{\circ}$$

The road adhesion condition determines the range of vehicle lateral force that can be provided, and it also affects vehicle control stability. The following constraints should be met between the vehicle lateral acceleration and the road adhesion condition:

(30) $$-\mu g\leq a_{y}\leq \mu g$$

Therefore, the specific optimization problem can be equivalent to the multi-constraint quadratic programming problem, which can be expressed as:

(31) $$\{\begin{array}{c} \begin{array}{c} min\overset{N_{cp}}{\underset{i=1}{\sum }}\|\eta_{c}\left(k+i|t\right)-\eta_{pref}{\left(k+i|t\right)\|}_{Q_{c}}^{2}+\overset{N_{cc}-1}{\underset{i=1}{\sum }}\|\Delta U{\left(k+i|t\right)\|}_{R_{c}}^{2}+\rho \varepsilon^{2}\\ st.\;\;\;\;-25{}^{\circ}\leq \delta_{f}\leq 25{}^{\circ} \end{array}\\ -0.47{}^{\circ}\leq \Delta \delta_{f}\leq 0.47{}^{\circ}\\ \begin{array}{c} -6{}^{\circ}<\alpha_{f}<6{}^{\circ}\\ \begin{array}{c} -\mu g\leq a_{y}\leq \mu g\\ \varepsilon >0 \end{array} \end{array} \end{array}$$

By solving Equation (32), the increment sequence of the control quantity can be expressed as:

(32) $$\Delta U\left(t\right)=\left[\begin{array}{c} \Delta u\left(t|t\right)\\ \Delta u\left(t+1|t\right)\\ \vdots \\ \Delta u\left(t+N_{c}-1|t\right) \end{array}\right]$$

On this basis, the first increment of control quantity in Equation (33) is taken as the actual output and is superimposed with the actual output control quantity in the previous period to obtain the actual control output quantity in the current period:

(33) u(t)=u(t−1)+Δu(t|t)

The actual output control quantity was implemented on the system, and the objective function was resolved based on the feedback state quantity in the next control cycle. Therefore, the incremental sequence of the control quantity was constantly updated to achieve the purpose of rolling optimization. Finally, the above optimization solution process was repeated to complete the vehicle trajectory tracking control. 

## 4. Driving Simulator Experiments

To make the trajectory planned by the obstacle avoidance trajectory planning controller satisfy the safety requirements and be more human-like, it is necessary to extract the human driver’s obstacle avoidance trajectory and perform statistical analysis on the trajectory characteristics, which provides a basis for the parameters design of the trajectory planning controller. Therefore, in this study, the obstacle avoidance experiments based on a driving simulator with six degrees of freedom were implemented, and the obstacle avoidance trajectories from different drivers were extracted for further analysis. 

### 4.1. Apparatus

**Figure 7:**
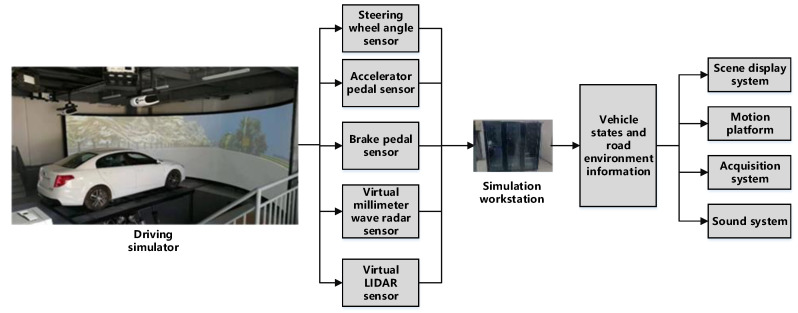
Composition of the driving simulator.

Considering that the actual vehicle obstacle avoidance experiment possesses certain risks, this study employed a driving simulator to perform the obstacle avoidance experiment. The driving simulator is a modified simulation technology that combines pure digital simulation with field test. The vehicle, driving field, and various types of sensors are constructed by a digital method to reproduce the real driving scene and satisfy various requirements of the vehicle test and development. The driving simulator tests are characterized by low cost, high efficiency, repeatability, and low risk coefficient. The driving simulator used in this work is presented in Figure 7. The simulator mainly includes a vibration platform with six degrees of freedom, a front view ring display system, a cockpit system, and high-performance workstation, which has a strong sense of immersion in driving operation. In addition, the driving simulator is equipped with multiple sensors for collecting the driver’s operation and road environment information, including steering wheel angle sensor, accelerator pedal sensor, brake pedal sensor, virtual millimeter wave radar sensor, and virtual LIDAR sensor. 

### 4.2. Participants and Experimental Program

Twenty-eight experienced drivers participated in the obstacle avoidance experiment. The ages of the drivers ranged from 23 to 48 years old, with an average age of 32.2 years (standard deviation = 5.82). Their driving experience ranged from 5 to 26 years (mean = 12.6, standard deviation = 4.6). All of the participants were non-professional drivers with a valid driver’s license, normal or corrected vision, and who had experienced no serious traffic accidents over the past three years. 

**Figure 8:**
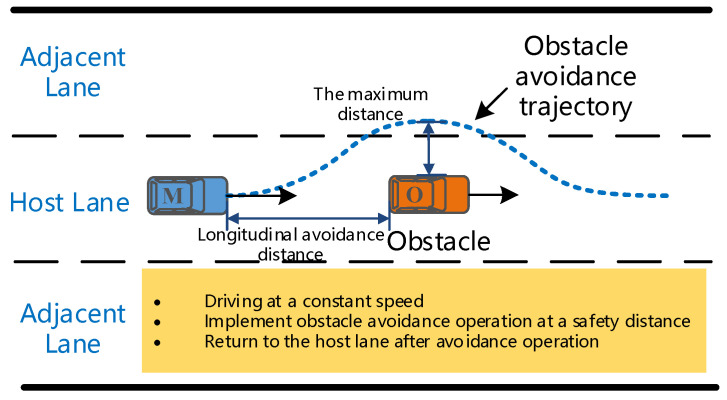
Schematic diagram of the obstacle avoidance experiment.

A two-way six-lane straight urban road with a length of 2 km was selected as the test section to implement the obstacle avoidance experiment, as exhibited in Figure 8. The obstacle was stationary and placed in the middle lane 1 km from the vehicle starting point. Each participant was required to navigate the vehicle at three speeds of 40 km/h, 60 km/h, and 80 km/h from the starting point, and drove forward along the center line of the middle lane. The participants were required to execute the obstacle avoidance operation in a safety distance according to their driving habits when they noticed the obstacle in front of the road. They were also required to return to the original lane after the completion of the obstacle avoidance operation. The size of the obstacle was 4710 × 1820 × 1500 mm. Each participant needed to complete three tests at different speeds and try to keep a constant speed during the avoidance operation. 

### 4.3. Procedures

Before the experiment, the drivers were asked to participate in a practice round for approximately 10 min to familiarize themselves with the driving simulator and testing process. Next, the test staff introduced the experimental objectives and notes. After the beginning of the experiment, the participants performed the obstacle avoidance operation as required, and relevant data would be recorded in real time. After each experiment, the participants were free to manipulate the driving simulator until the beginning of the next experiment. To alleviate driving fatigue, the participants could rest for 5 min after every testing period. During the test, the driver was required to strictly abide by the traffic rules. In case of emergency, such as the abnormal operation of the driving simulator or equipment, the unsatisfactory condition of the participants, and so on, the test would be stopped immediately and the test vehicle would be safely parked in the emergency parking zone. Participants were paid ¥100 for their participation after they had finished all the experiments.

### 4.4. Collected Data

**Figure 9:**
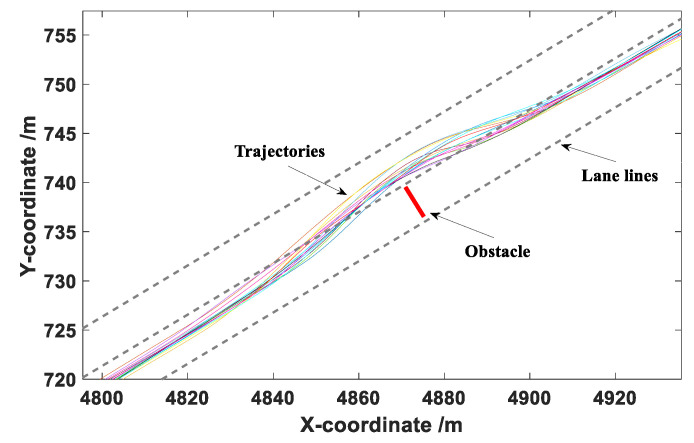
Obstacle avoidance trajectories under a vehicle speed of 40 km/h.

**Figure 10:**
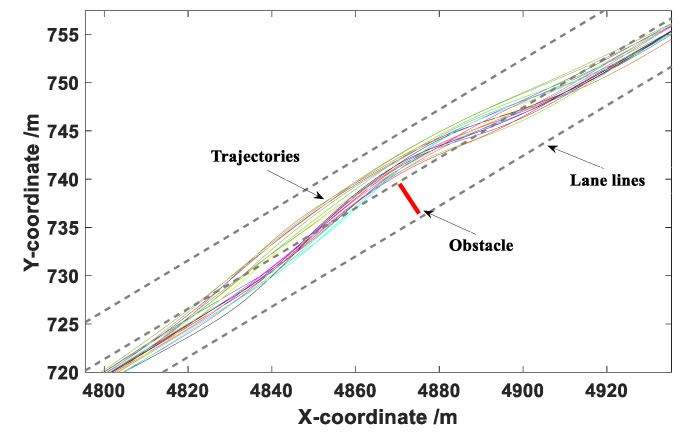
Obstacle avoidance trajectories under a vehicle speed of 60 km/h.

**Figure 11:**
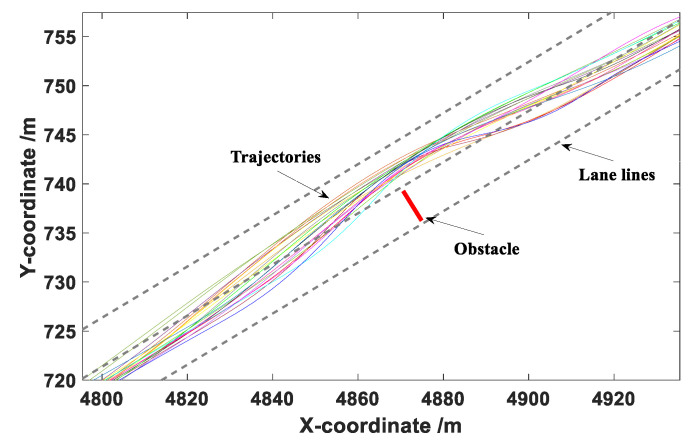
Obstacle avoidance trajectories under a vehicle speed of 80 km/h.

The data collected during the obstacle avoidance experiments mainly included the longitudinal and lateral coordinates of the vehicle in the road coordinate system, vehicle speed, and acceleration. The sampling frequency was 100 Hz. After the test, a total of 180 groups of effective obstacle avoidance data were obtained. Then, Matlab was used to fit the collected trajectories, with the results presented in Figure 9, Figure 10 and Figure 11. 

It can be seen from Figure 9, Figure 10 and Figure 11 that the drivers in each group of tests successfully completed the obstacle avoidance operation and the obstacle avoidance trajectory was smooth, so the data collected in the test were valid data. The longitudinal distance at the beginning of obstacle avoidance and the maximum lateral distance during the obstacle avoidance were statistically analyzed under different vehicle speeds. 

**Figure 12:**
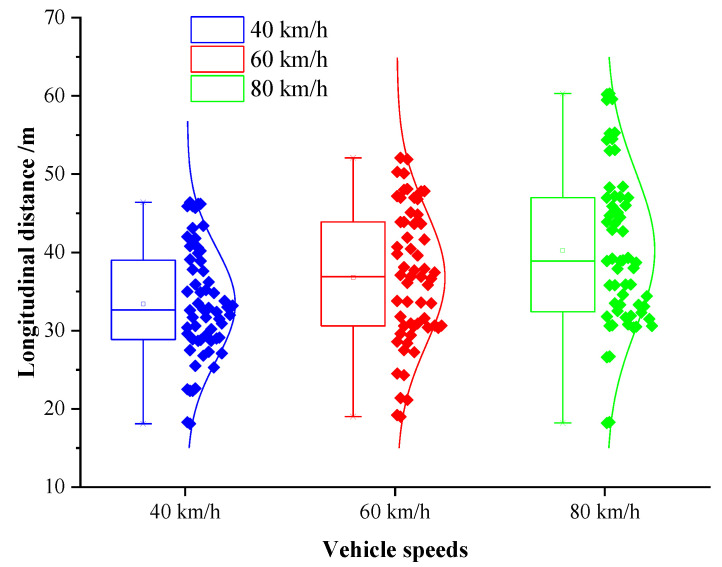
Box diagram of the longitudinal distance at the beginning of avoidance under different speeds.

The coordinate point when the vehicle generated continuous lateral displacement was determined as the starting position of the obstacle avoidance operation, and the distance between the starting point and the centroid of the obstacle was defined as the longitudinal distance at the beginning of obstacle avoidance. This value can provide a basis for the determination of the A value in the elliptical repulsive potential field (shown in Figure 4). The box diagram of longitudinal distance at the beginning of obstacle avoidance under different vehicle speeds is presented in Figure 12. 

It can be seen from Figure 12 that the average longitudinal distance at the beginning of obstacle avoidance under the speeds of 40 km/h, 60 km/h, and 80 km/h were 33.4 m, 37.5 m, and 40.6 m, respectively, and the medians were 32.7 m, 37.0 m, and 38.1 m. The longitudinal distance increased with the promotion of the vehicle speed. The results of the one-way analysis of variance indicated that the vehicle speed possessed a significant effect on the longitudinal distance at the beginning of obstacle avoidance (p=0.000<0.05, F(2,177)=9.320). Therefore, in this paper, the vehicle speed and the longitudinal distance were determined as reference factors, and the least square method was used for linear regression fitting. The expression is as follows:

(34) $$a=0.1725v_{p}+26.517$$

where a is the longitudinal distance at the beginning obstacle avoidance, and $v_{p}$ is the vehicle speed. 

**Figure 13:**
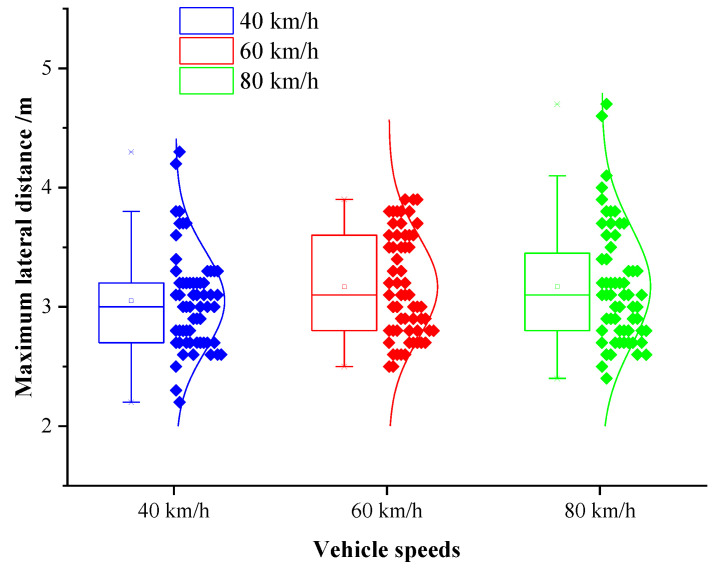
Box diagram of the maximum lateral distance under different speeds.

The maximum lateral distance was defined as the maximum lateral distance between the vehicle and the obstacle during the obstacle avoidance process. This value can provide a basis for the determination of the B value in the elliptical repulsive potential field (shown in Figure 4). The box diagram of the maximum lateral distance under different vehicle speeds is presented in Figure 13.

It can be seen from Figure 13 that the average maximum lateral distance during the process of obstacle avoidance under the speeds of 40 km/h, 60 km/h, and 80 km/h were 3.44 m, 3.57 m, and 3.65 m, respectively, and the medians were 3.51 m, 3.63 m, and 3.71 m. The maximum lateral distance increased slightly with the promotion of the vehicle speed. The results of the one-way analysis of variance indicated that the vehicle speed possessed no significant effect on the maximum lateral distance during the process of obstacle avoidance (p=0.254>0.05, F(2,177)=1.380). Therefore, in this paper, the average of the maximum lateral distance of all data was determined as the final value of maximum lateral distance:

(35) b=3.46 m

where b is the maximum lateral distance.

## 5. Co-Simulation Results Analysis

### 5.1. Co-Simulation Model Establishment

**Figure 14:**
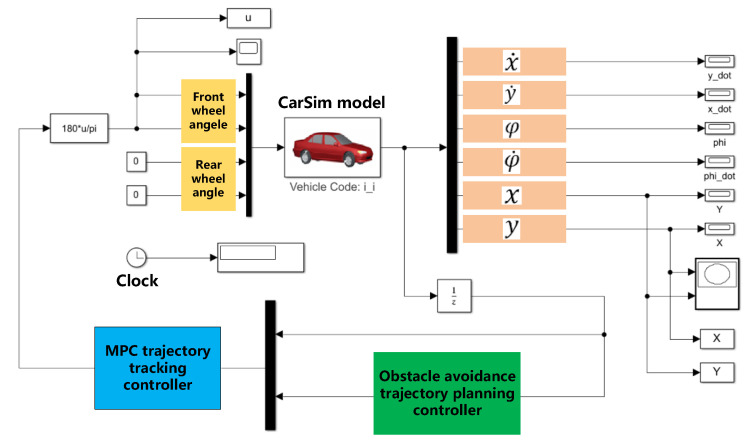
CarSim/Simulink co-simulation model.

To verify the obstacle avoidance trajectory planning controller and the MPC trajectory tracking controller designed in this study, a co-simulation model based on CarSim and Simulink was established for simulation testing. The co-simulation model is illustrated in Figure 14. 

As shown in Figure 14, $\dot{x}$ is the vehicle longitudinal speed, $\dot{y}$ is the vehicle lateral speed, φ is the vehicle heading angle, $\dot{\varphi }$ is the vehicle yaw rate, and x and y are the vehicle coordinate information in the geodetic coordinate system. CarSim was responsible for building the vehicle dynamics model, as the Vehicle Code: i_i module shown in the figure, and outputting the coordinate information, the longitudinal and lateral speeds, the heading angle and the yaw rate to the trajectory planning controller and the trajectory tracking controller, respectively. Simulink was responsible for constructing the trajectory planning model based on the modified APF algorithm and the trajectory tracking model based on the MPC algorithm. The trajectory planning controller provided a reference trajectory for the trajectory tracking controller, and the tracking module outputted the final calculated front wheel angle to the vehicle dynamics module in CarSim. Then, the updated vehicle state parameters were employed for calculation in the next control period. 

The B-Class Hatchback with front-wheel drive was selected as the vehicle dynamics simulation model in CarSim, and the main parameters are shown in Table 1. 

The specific simulation conditions were set as follows: the global reference trajectory was a straight path; the road adhesion coefficient was set as 0.8; the obstacle coordinate was set as (105, 0), and the obstacle was 4710 × 1820 × 1500 mm, and the vehicle speeds were 40 km/h, 60 km/h, and 80 km/h respectively. 

The specific parameters of the trajectory planning controller and trajectory tracking controller in Simulink were set as follows: the prediction step size and control step size of the trajectory planning controller were determined as $N_{pp}=15$, and $N_{pc}=5$; the weight matrixes of the trajectory planning controller were determined as $Q_{p}=\left[\begin{array}{ccc} 100 & 0 & 0\\ 0 & 100 & 0\\ 0 & 0 & 100 \end{array}\right]$, $R_{p}=10$; the prediction step size and control step size of the trajectory tracking controller were determined as $N_{cp}=20$ and $N_{cc}=10$; and the weight matrixes of the trajectory planning controller were determined as $Q_{p}=\left[\begin{array}{ccc} 2000 & 0 & 0\\ 0 & 1000 & 0\\ 0 & 0 & 1000 \end{array}\right]$, $R_{p}=1.5\times 10^{5}$. The control period of both controllers was 0.01 s.

### 5.2. Co-Simulation Results

**Figure 15:**
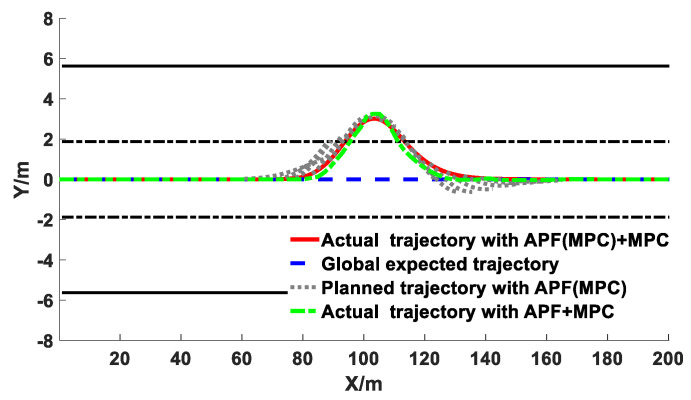
Co-simulation results of obstacle avoidance trajectory planning under 40 km/h.

**Figure 16:**
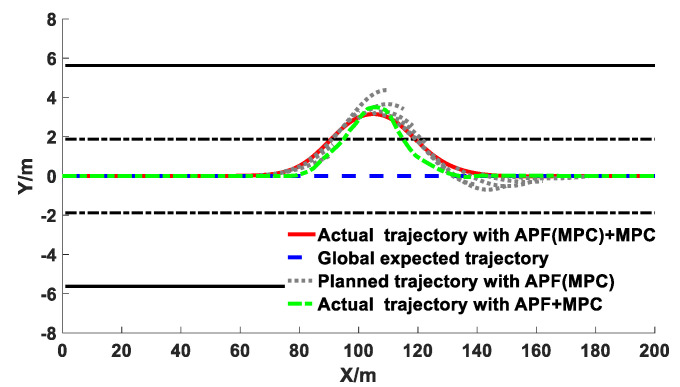
Co-simulation results of obstacle avoidance trajectory planning under 60 km/h.

**Figure 17:**
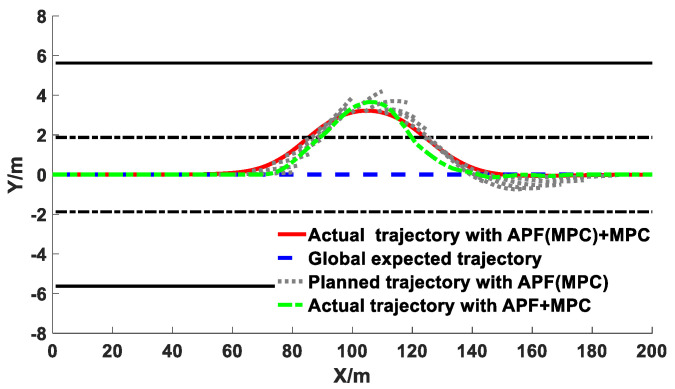
Co-simulation results of obstacle avoidance trajectory planning under 80 km/h.

The comparison results of the co-simulation of the obstacle avoidance trajectory planning with different algorithms under different vehicle speeds are exhibited in Figure 15, Figure 16 and Figure 17. 

**Figure 18:**
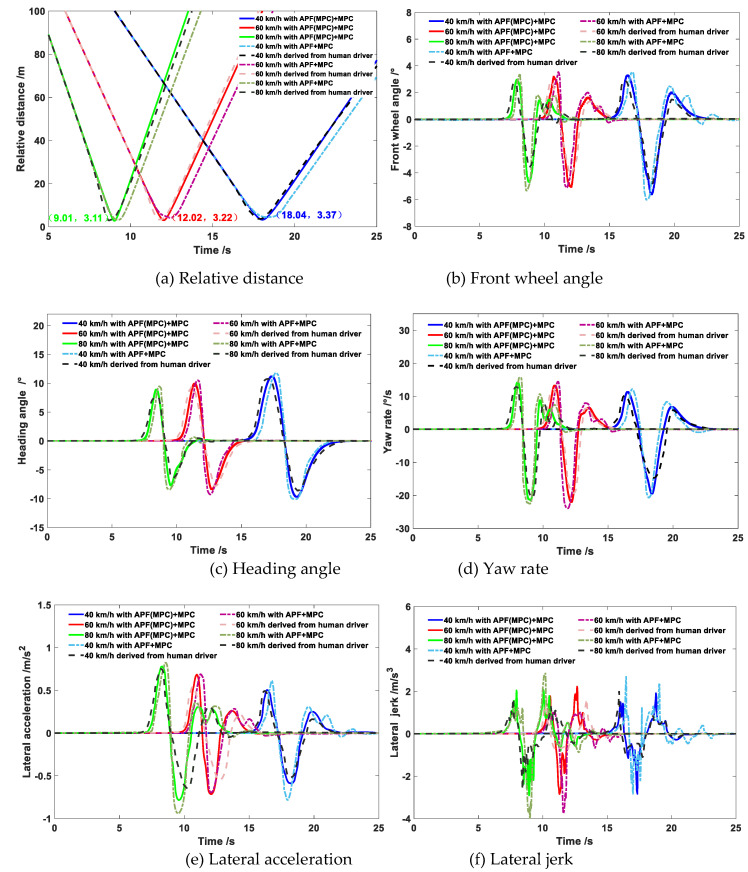
Co-simulation results of vehicle states during the obstacle avoidance process under different speeds.

It can be observed from Figure 15, Figure 16 and Figure 17 that under different vehicle speeds, the obstacle avoidance controllers based on the APF with MPC model derived from previous study [41] and the APF(MPC) with MPC model proposed in this study can effectively plan and track the local obstacle avoidance trajectory that satisfies the obstacle and road boundary constraints in real time in the predicted time domain, and the trajectory tracking controllers based on the linear time-varying MPC algorithm can promptly and accurately track the first two reference points of the local planned trajectory from the trajectory planning controllers in real time. The vehicles avoided obstacles smoothly under different speeds, which indicated that the trajectory planning and tracking controllers were both feasible and effective. However, under different vehicle speeds, the longitudinal distance at the beginning of the obstacle avoidance derived from the controller proposed in this study were larger than that of resulting from the other controller (APF with MPC). The longitudinal distance from the proposed model in this study under the vehicle speeds of 40 km/h, 60 km/h, and 80 km/h were 34 m, 38 m, and 41 m, respectively; while the values from the previous model were 29 m, 34 m, and 37 m, respectively. With the increase of vehicle speed, the obstacle avoidance trajectory planning was advanced and the longitudinal distance was promoted. In addition, the maximum lateral distance during the obstacle avoidance process remained basically unchanged, and the value under the different vehicle speeds from the proposed model in this study were 3.48 m, 3.50 m, and 3.51 m, respectively, while the values from the previous model were 3.69 m, 3.77 m, and 3.87 m, respectively. The specific results during the process of the obstacle avoidance control are exhibited in Table 2 and Figure 18. 

As shown in Table 2, under different vehicle speeds, the maximum values of the front wheel angle, heading angle, yaw rate, lateral acceleration, and lateral jerk during the obstacle avoidance trajectory tracking process derived from the previous study model (APF+MPC) were obviously greater than that of derived from the proposed model in this study and human drivers. Since the prediction time domain cannot be designed too large in the APF with MPC model, the obstacle avoidance trajectory would possess a smaller longitudinal distance and a larger lateral distance, which would affect the smoothness of the trajectory tracking process. Similarly, larger maximum values of the lateral acceleration and lateral jerk would also reduce the passenger’s comfort. Since the APF(MPC) with MPC model proposed in this study combined the APF and MPC in the trajectory planning layer, the trajectory planning controller would take into account the vehicle kinematics constraints in advance, and the additional MPC was equivalent to further improving the model prediction time domain, so that the controller can better simulate the driver’s preview behavior. Moreover, too many complex constraints often made it impossible for MPC controller to obtain the optimal solution. The additional MPC in the planning layer could relieve the computational pressure of the MPC algorithm in the trajectory tracking layer. The kinematics and other constraints of the vehicle had been taken into account during the trajectory planning process, and the MPC in the tracking layer can focus on solving the vehicle dynamics constraints, which can improve the effectiveness of the controller in solving the optimal value. Therefore, on the one hand, the results of the longitudinal distance and maximum lateral distance derived from the controller designed in this study were more in accordance with human driver’s obstacle avoidance trajectory characteristics in Section 4, and on the other hand, the results of the front wheel angle, heading angle, yaw rate, lateral acceleration, and lateral jerk during the trajectory tracking process derived from the proposed model in this study were more smooth and more human-like, which can effectively improve the acceptance of the autonomous driving system or the intelligent driving system.

The comparison results of the relative distance between the vehicle and obstacle, front wheel angle, heading angle, yaw rate, lateral acceleration, and lateral jerk derived from the APF with MPC model, APF(MPC) with MPC model, and human drivers during the obstacle avoidance process are presented in Figure 18. 

As shown in Figure 18a, the minimum distance between vehicle and obstacle derived from the APF(MPC) with MPC model under the speeds of 40 km/h, 60 km/h, and 80 km/h were 3.37 m, 3.22 m, and 3.11 m, respectively; the value from the APF with MPC model were 3.34 m, 3.18 m, and 3.10 m, respectively; the value from the human driver were 3.36 m, 3.21 m, and 3.11 m, respectively. The minimum distances from different models under different speeds were all greater than the safe distance of 2.8 m (the distance of vehicle mass center to the right front corner added the distance of obstacle mass center to the left rear corner), which indicated that the vehicle would keep a reasonably safe distance from the obstacle during the obstacle avoidance process. As shown in Figure 18b–d, the front wheel angle derived from the APF(MPC) with MPC model under all of the different speeds did not exceed 6°, which satisfied the kinematic constraints of the vehicle. The front wheel angle and heading angle decreased with the increase of the vehicle speed, which ensured the smoothness and comfort of the obstacle avoidance process during high speed driving. The range of yaw rate was basically consistent under different speeds, and all of them satisfied the requirements of comfort. However, during the process of changing back to the middle lane, the front wheel angle and yaw rate derived from the APF with MPC model would produce slight vibrations, which would affect the smoothness of the obstacle avoidance trajectory. As shown in Figure 18e,f, the lateral acceleration and lateral jerk improved with the increase of the vehicle speed. Since the longitudinal distance at the beginning of the obstacle avoidance derived from the APF with MPC model was the smallest, the maximum acceleration was the largest and the acceleration changed dramatically, which would affect the smoothness and comfort. In summary, the trajectory planning and tracking controllers designed in this work can satisfy the static obstacle avoidance requirements at different speeds. The variations of the relevant parameters during the obstacle avoidance process were more human-like, and the avoidance operation was completed on the premise of ensuring smoothness and comfort. 

**Figure 19:**
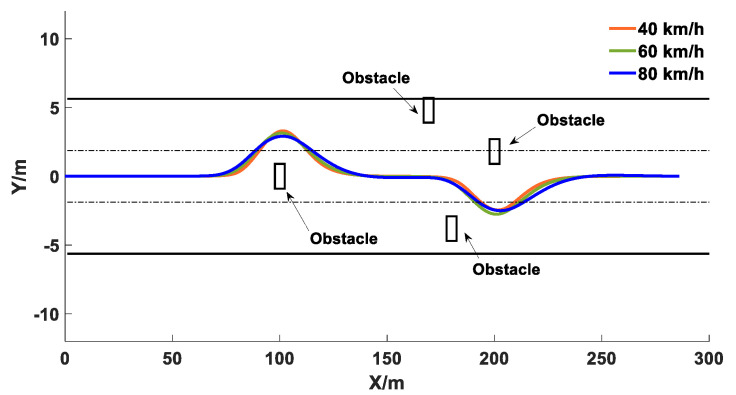
Co-simulation results of obstacle avoidance trajectory tracking under different speeds.

The simulation results with multiple obstacles are presented in Figure 19. The coordinates of the obstacles are (100, 0), (160, 4), (170, −3.75), and (200, 1.8), respectively. 

As shown in Figure 19, under different vehicle speeds, the proposed obstacle avoidance controller successfully achieved the goal of avoiding multiple obstacles, and the actual trajectories were smooth and continuous. In addition, there was no phenomenon that the vehicle fell into a local minimum point and the target was unreachable. Therefore, the co-simulation results demonstrated that the proposed trajectory planning controller and the trajectory tracking controller can effectively ensure the safety of obstacle avoidance operations. 

## 6. Conclusions

In this work, an obstacle avoidance trajectory planning controller based on a modified APF algorithm and the MPC algorithm and a trajectory tracking controller based on the linear time-varying MPC algorithm were designed for the AV to realize the active obstacle avoidance function. The modified APF model proposed in this paper added a road boundary repulsive potential field and ameliorated the obstacle repulsive potential field based on the traditional APF model, overcoming some defects of the traditional model. To make the modified APF model satisfy the kinematic constraints of the vehicle, the MPC algorithm was combined with the modified APF model, and a reasonable objective function was constructed to minimize the deviation between the planning trajectory of the modified APF model and the predicted trajectory of the MPC algorithm. Considering that there were many kinds of constraints during vehicle lateral control and for the sake of guaranteeing real-time capability, accuracy, and robustness of the trajectory tracking control algorithm at different speeds, a linear time-varying model predictive trajectory tracking controller was established on the basis of linearizing the vehicle monorail dynamic model. The controller determined the vehicle front wheel angle as the control variable, and multiple constraints of vehicle dynamics and kinematics were combined to design the objective function that could achieve the requirements of fast and accurate tracking of the desired trajectory.

Ameliorating the human-like degree of the planning trajectory is the core of improving the acceptance of the autonomous driving system. Therefore, in this study, a human driver’s obstacle avoidance experiment was implemented based on a six-degree-of-freedom driving simulator equipped with multiple sensors, including a steering wheel angle sensor, accelerator pedal sensor, brake pedal sensor, virtual millimeter wave radar sensor, and virtual LIDAR sensor. The obstacle avoidance trajectories under different speeds from different drivers were collected, and the longitudinal distance at the beginning of the obstacle avoidance operation and the maximum distance during the obstacle avoidance process underwent statistical analysis. These two parameters can provide a basis for the determination of the A value and B value in the elliptical repulsive potential field (shown in Figure 4), making the planned trajectory more human-like. 

Finally, a co-simulation model based on CarSim/Simulink was established for the off-line simulation testing of the obstacle avoidance trajectory planning controller and the trajectory tracking controller designed in this study. The co-simulation results demonstrated that the vehicles could smoothly avoid obstacles under different speeds. The results of relevant parameters during the obstacle avoidance process were in accordance with the human drivers’ obstacle avoidance trajectory characteristics in Section 4, which indicated that the proposed trajectory planning controller and the trajectory tracking controller were more human-like under the premise of ensuring the safety and comfort of the obstacle avoidance operation. 

A few deficiencies in this study need to be improved in the future work. Different road environments may have an impact on driver’s obstacle avoidance behavior. A future study will pay close attention to collect the driver’s operation data under different road environments and analyze the difference. In addition, the parameters of the obstacle avoidance controller in complex scenarios need to be further optimized. 

## Figures and Tables

**Table 1:** Basic parameters of the vehicle dynamics model.

Symbol.	Parameters	Value
*m*	Vehicle sprung mass	1563.1 kg
*l_f_*	Distance between the center of mass and the front axis	1174.2 mm
*l_r_*	Distance between the center of mass and the rear axis	1358.8 mm
*h_g_*	Height of the center of mass	716.5 mm
*B*	Wheel track	1480 mm
*f*	Coefficient of rolling resistance	0.015
*C_D_*	Coefficient of air resistance	0.8

**Table 2:** Comparison results of performance parameters.

Models	Speed	$\left|\delta_{f,max}\right|$	$\left|\varphi_{max}\right|$	$\left|{\dot{\varphi }}_{max}\right|$	$\left|a_{y,max}\right|$	$\left|j_{y,max}\right|$
APF+MPC	40 km/h	6.02°	11.81°	20.91°/s	0.79 m/s^2^	3.34 m/s^3^
	60 km/h	5.21°	10.51°	23.94°/s	0.72 m/s^2^	3.80 m/s^3^
	80 km/h	5.35°	9.46°	23.58°/s	0.95 m/s^2^	3.84 m/s^3^
APF(MPC)+MPC	40 km/h	5.73°	11.14°	18.69°/s	0.59m/s^2^	2.82 m/s^3^
	60 km/h	4.92°	9.82°	21.17°/s	0.70 m/s^2^	2.86 m/s^3^
	80 km/h	4.88°	8.79°	21.19°/s	0.79 m/s^2^	2.91 m/s^3^
Human driver	40 km/h	4.85°	10.77°	14.74°/s	0.52 m/s^2^	2.31 m/s^3^
	60 km/h	4.24°	9.36°	19.44°/s	0.69 m/s^2^	2.04 m/s^3^
	80km/h	3.62°	7.68°	20.91°/s	0.71 m/s^2^	2.85 m/s^3^
